# Serological Survey for Avian Influenza in Turkeys in Three States of Southwest Nigeria

**DOI:** 10.1155/2015/787890

**Published:** 2015-11-17

**Authors:** Daniel Oladimeji Oluwayelu, Comfort Oluladun Aiki-Raji, Oladunni Taiwo Adigun, Opeyemi Kazeem Olofintuyi, Adebowale Idris Adebiyi

**Affiliations:** Department of Veterinary Microbiology and Parasitology, University of Ibadan, Ibadan 20005, Nigeria

## Abstract

Since the first outbreak of avian influenza (AI) in Nigeria in 2006, there has been continuous monitoring of the disease in chickens with little attention given to turkeys. As part of on-going surveillance for AI in southwest Nigeria, we used a competitive ELISA to detect anti-AI virus antibodies in 520 turkey sera obtained from poultry farms in Oyo, Osun, and Ondo states while haemagglutination inhibiting antibodies against low pathogenic AI viruses (LPAIVs) were detected using H3N8 and H5N2 subtype-specific antigens. The overall seroprevalence obtained by ELISA was 4.4% (23/520). Of the 23 ELISA-positive samples, 18 were positive for anti-AIV H3N8 antibodies only and four were positive for both anti-AIV H3N8 and H5N2 antibodies indicating a mixed infection, while five were negative for antibodies to either of the two AIV subtypes. Considering that turkeys have been implicated as a mixing vessel for generating influenza virus reassortants of human and avian origin, the detection of antibodies to LPAIV H3N8 and H5N2 in these turkeys is of public health concern. We advocate further studies to determine the potential role of turkeys in the zoonotic transmission of AIVs in Nigeria. Additionally, the practice of rearing turkeys with chickens should be discouraged.

## 1. Introduction

Avian influenza (AI) is a highly contagious disease caused by type A influenza viruses, a member of the family Orthomyxoviridae [1]. Influenza A viruses have antigenically related nucleocapsid and matrix proteins but are classified into subtypes on the basis of their haemagglutinin (H) and neuraminidase (N) antigens. Although Fouchier et al. [2] reported the presence of 16 H and 9 N subtypes of influenza A viruses, recent studies [3, 4] have identified additional H17N10 and H18N11 subtypes in bats. Many species of domestic and wild birds worldwide have been shown to be susceptible to infection with avian influenza viruses (AIVs), with aquatic birds constituting a major reservoir of these viruses which have particularly been reported to occur in poultry in either the highly pathogenic or low pathogenic forms; the overwhelming majority of isolates are of low pathogenicity for chickens and turkeys [5, 6]. Specifically, turkeys are susceptible to a wide range of influenza A viruses and, as a major exception to the host range restriction rule, are routinely infected with swine-like influenza viruses [7]. Infections in turkeys range from asymptomatic to severe disease including respiratory tract disease, depression, drop in egg production, and high mortality [7, 8].

The segmented nature of the influenza virus genome allows for reassortment of genes when a susceptible host is coinfected with different influenza virus subtypes [9], which may be from different species. This interspecies transmission of influenza viruses has been reported by several authors [9–12]. Influenza viruses with novel combinations of gene segments from different influenza-susceptible species have been isolated from turkeys [12, 13], which have been indicated to be more susceptible to AI infections than chickens [14]. Although H3- and H5-subtype influenza viruses are known to infect avian and mammalian species, including humans [15, 16], the H3-subtype viruses are usually not the subject of conventional surveillance, which is biased towards detection of highly pathogenic avian strains, therefore leading to fewer H3 virus isolations from poultry flocks.

Since 2005, AI has spread from Southeast Asia to over 60 different countries, resulting in the direct death or slaughter of over 250 million poultry [17]. Recently, an outbreak of highly pathogenic avian influenza (HPAI) H5N2 that affected mostly turkey flocks was reported in several counties in Minnesota, USA [18, 19]. In Nigeria which has a poultry industry of about 160 million birds estimated at US$ 250 million [20], AI has been reported in several domestic and wild birds such as chickens [21], ducks [22], waterfowls [23], and spur-winged geese or whistling ducks [24]. Although these studies emphasize the importance of surveillance for AIV infections in the natural hosts, there has been little or no report of surveillance for the disease in turkeys, which form almost 2% of the total poultry population, in Nigeria [25].

According to the OIE [6], in serologic surveillance programs, the test to detect the anti-nucleoprotein antibody is the method commonly used because it detects antibodies to a cross-reactive antigen shared by all influenza A viruses. Some authors [26, 27] have suggested that competitive enzyme-linked immunosorbent assay (cELISA) is effective for large-scale surveillance of AIV in avian flocks or herds of other species and this test has been used to detect antibodies against different AIV strains, including H3N8 and H5N2, in chickens, ducks, turkeys, and other avian species [27]. These authors reported that since the cELISA is high in sensitivity but relatively low in specificity in turkeys, quails, and pheasants, its use for surveillance purposes should be followed by a conventional standard test (e.g., haemagglutination inhibition assay) when specific species need to be tested [27, 28]. Therefore, as part of on-going surveillance for AI in poultry, we carried out a serologic survey to investigate the prevalence of avian H3- and H5-subtype influenza virus antibodies in turkey flocks in three states of southwest Nigeria.

## 2. Materials and Methods

### 2.1. Study Area and Survey Animals

**Figure 1:**
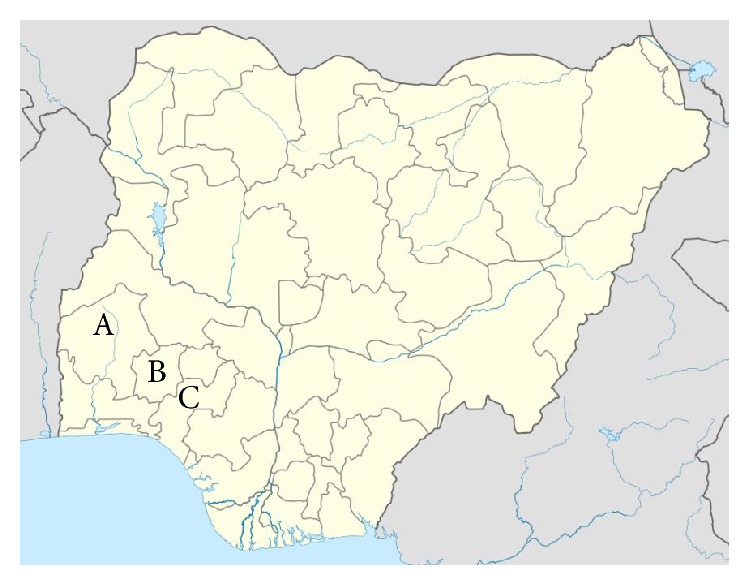
Map of Nigeria showing the study area (A = Oyo; B = Osun; C = Ondo).

A total of 520 (203 males and 317 females) apparently healthy turkeys sampled from 22 different flocks located in Oyo, Osun, and Ondo states, southwest Nigeria (Figure 1), were used for this study. While 315 samples were collected from 13 flocks in Oyo state, 95 were collected from six flocks in Osun state and 110 samples were collected from only three flocks in Ondo state. Additionally, the turkeys comprised 331 local and 189 exotic varieties. The southwest region plays a leading role in poultry production since an estimated 65% of Nigeria's commercial poultry population is concentrated there [25, 29].

### 2.2. Sample Collection

About 2 mL of blood was aseptically collected from the brachial vein of each turkey using sterile syringes and needles. The blood was allowed to clot at room temperature while sera were separated and stored at −20°C until tested. The farmers were interviewed on issues such as AI vaccination of their flocks and type of poultry they preferred to rear while the practices they employed in rearing the turkeys were observed. Additionally, the sex and breed of the turkeys as well as management system (free-range, semi-intensive, or intensive) were recorded. The turkeys were grouped based on age into growers (0–18 weeks) and adults (>18 weeks).

### 2.3. Serology

A competitive ELISA kit (BioNote Inc., Korea) for the quantitative detection of anti-nucleoprotein antibodies to AIV was used to screen the sera. According to the manufacturer, this kit was validated using antisera against H1–H15 subtypes of AIV. The test was performed following the kit protocol and results were read at 450 nm using a microplate ELISA reader (Optic Ivymen System, Model 2100C, Biotech SL, Madrid, Spain). The percentage inhibition (PI) for each sample was calculated from the absorbance values obtained. Positive samples (PI value ≥ 85) were screened by haemagglutination inhibition (HI) test for AIV subtype-specific antibodies using a panel of reference antigens comprising low pathogenic avian influenza (LPAI) H3N8 and H5N2 viruses and 4 haemagglutinating units of each antigen according to standard protocol [6]. Data obtained were analysed with column statistics and Fisher's exact test (two-tailed) using Graph Pad prism version 5.0 (Graph Pad software, San Diego, CA, USA) and *p* values < 0.05 were considered significant.

## 3. Results and Discussion

**Figure 2:**
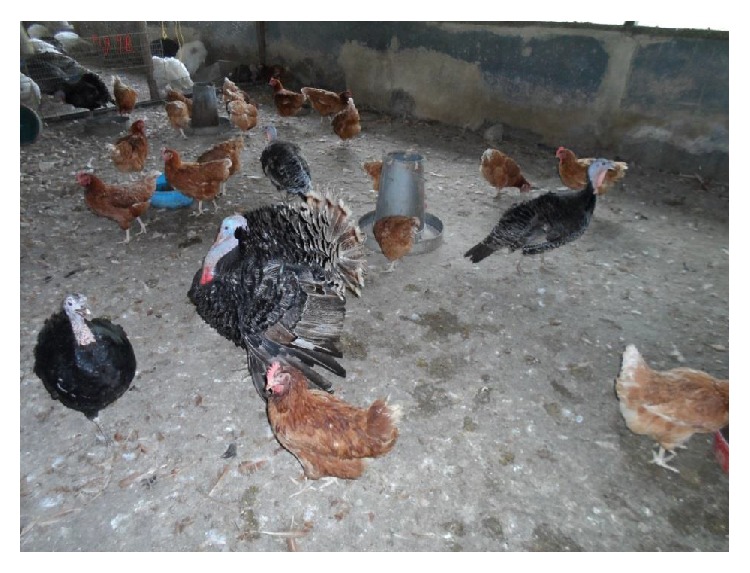
Practice of rearing turkeys with chickens.

Interviews conducted with the farmers revealed that the sampled turkeys were not vaccinated against AI and rearing of turkeys with chickens was a common practice on some of the farms (Figure 2). It was also observed in two farms (one each in Oyo and Osun states) that the turkeys were kept in close proximity to pig pens while the farmers interviewed in Ondo state indicated their preference for rearing chickens instead of turkeys. Based on the ELISA, prevalence of anti-AIV antibodies in the tested turkey sera was 6.0% (19/315), 4.2% (4/95), and 0% (0/110) for Oyo, Osun, and Ondo states, respectively, with overall seroprevalence of 4.4% (23/520). Compared to the intensively raised turkeys, those reared on free-range system had significantly higher AIV antibody prevalence (Table 1), with *p* value of 0.034 and odds ratio (OR) of 9.4 (95% CI: 1.7–51). However, there was no significant difference in seropositivity based on state, breed, age, and sex of the birds although a greater proportion of the HI test-positive birds were intensively reared adult, female local turkeys from Oyo state (Table 2). The HI antibody titres ranged from 1 : 8 to 1 : 2048 and 1 : 64 to 1 : 2048 for LPAIV H3N8 and H5N2 subtypes, respectively. Of the tested sera, 18 were positive for anti-AIV H3N8 antibodies only and four were positive for both anti-AIV H3N8 and H5N2 antibodies, while five did not contain antibodies to either of the two AIV subtypes. Mean HI antibody titres of 5.4 ± 0.6 log_2_ (95% CI: 4.1–6.8) and 9.8 ± 1.3 log_2_ (95% CI: 5.8–13.7) were obtained for the H3N8- and H5N2-positive sera, respectively.

The current global influenza situation is characterized by a number of trends that must be closely monitored. These include an increase in the variety of animal influenza viruses cocirculating and exchanging genetic material, giving rise to novel strains [30]. Specifically, avian influenza viruses continue to be a problem worldwide because they are potentially highly infectious and can rapidly spread and cause disease in domestic poultry, and some may also infect other animal hosts, including humans [31]. Moreover, Clark and Hall [14] noted that the first sign of LPAI infection in domestic poultry is often seroconversion, which may be the only evidence of infection with some LPAI subtypes (i.e., no clinical signs present). These observations, coupled with previous reports of AIV infections in Nigeria [21–24, 32, 33], underscore the need for continuous surveillance for these infections in Nigerian poultry.

The detection of antibodies to LPAIV H3N8 and H5N2 in apparently healthy turkeys in this study indicates that LPAI H3N8 and H5N2 virus strains presently circulate in exotic and local turkey flocks in Oyo and Osun states, southwest Nigeria. Since vaccination against AI is not currently officially permitted in Nigeria and all the farmers interviewed in this study claimed that they did not vaccinate their birds against avian influenza, the antibodies detected in these birds could only have resulted from seroconversion following natural infection with the viruses. Thus, the birds could serve as reservoirs shedding the viruses into the environment, thereby playing a crucial role in the epidemiology of the disease. This finding is consistent with previous reports of infection with LPAIV H3 and H5 subtypes in poultry elsewhere [27, 34, 35] and corroborates the observation of Clark and Hall [14] that the first sign of LPAI infection in domestic poultry is often seroconversion, which may be the only evidence of infection. The nondetection of anti-AIV antibodies in turkey sera from Ondo state could be due to the fact that the samples were collected from only three flocks where the farmers interviewed indicated their preference for rearing chickens instead of turkeys.

Importantly, the detection of antibodies to both H3N8 and H5N2 LPAI viruses in some turkeys in this study is of public health concern because coinfection with different influenza viruses might provide the opportunity for reassortment, leading to the emergence of novel reassortant strains with zoonotic potential [30]. This finding is similar to that of Song et al. [27] who also detected antibodies against H3N8 and H5N2 AIV strains in chickens, turkeys and other avian species in Korea. Two of the farms (one each in Oyo and Osun states) from which seropositive birds were detected in this study also kept pigs in pens close to the turkey houses. According to Kapczynski et al. [36], coproduction of swine and turkeys on the same farm may increase the opportunity for reassortment between different AI and mammalian influenza viruses due to close contact between these species. Additionally, it has been reported [37] that if a field virus is a low pathogenic type, there is the risk of it mutating to a highly pathogenic form after circulating in susceptible poultry. Thus, the practice of rearing different animal species in the same pen or in close proximity, as observed in this study, may play a vital role in interspecies transmission of AIVs in Nigeria. It is interesting to note that five of the ELISA-positive sera did not contain antibodies to either H3N8 or H5N2 LPAI virus. This suggests the possibility of other AIV subtypes circulating among turkeys in the study area. There is therefore a need to further investigate AIV strains present in turkey flocks in southwest Nigeria using a larger panel of reference AIV subtypes as well as virus isolation and molecular identification techniques such as reverse transcriptase-polymerase chain reaction.

The detection of higher level of seropositivity to LPAI H3 virus in this study compared to the H5 subtype is significant because, unlike aquatic birds, poultry are not natural hosts of H3-subtype influenza viruses and, generally, H3-infected poultry are asymptomatic [16]. Furthermore, although the global focus remains on H5, H7, and H9 viruses as emerging disease threats for avian and mammalian species, the genetic and pathogenic changes found among H3 viruses [16] suggest a need to determine the current status of H3 AIV infections in poultry, especially among chickens and turkeys, which constitute the largest poultry species in Nigeria. Moreover, it is known that normal asymptomatic infection of avian species can silently maintain and transmit H3 influenza viruses provided there is an opportunity for genetic reassortment with other prevalent strains (e.g., H5 or H9) in avian populations [16], and this may be promoted where different avian species are kept together. Thus, mixed farming of turkeys and chickens, observed to be practiced on some farms in this study, is a critical management risk that should be discouraged. Since turkeys, like swine, have been implicated as another mixing vessel for generating influenza reassortants of human and avian origin in the field [36], additional studies are advocated to determine their potential role in the zoonotic transmission of AIV strains in Nigeria.

In this study, it was observed that the odds of detecting anti-AIV antibodies were 9.4 times higher in free-range than in intensively managed turkeys. This could be due to the fact that birds (and other animals) on free-range are often neglected and allowed to scavenge for food, thus exposing them to infectious agents. According to Alexander [5], most evidence obtained on the prevalence of influenza in different types of poultry and from different geographical locations supports the view that the primary introduction is from feral birds. Therefore, influenza viruses are most likely to infect poultry reared in a way that allows contact with feral birds, such as on free-range. Based on this, we recommend that free-range rearing of turkeys in the study area be discouraged as it has the potential to favour spread of AI infections between poultry populations. Additionally, in order to better understand the epidemiology of AI in southwest Nigeria, we propose the fact that future surveillance for the disease should be done in combination with innovative tools such as social network analysis and poultry market chain analysis, both of which have successfully been used elsewhere [38–41], for the control of within-country and transboundary livestock disease outbreaks. These innovations, which should involve stakeholders like the commercial and small-holder poultry farmers, poultry sellers and butchers, and live-bird market operators, have the benefits of being able to provide a network-based approach that offers new insights into disease transmission dynamics as well as an analytical framework that allows characterization of an entire poultry industry and interlinkages among various actors in the industry [38, 42, 43]. Ultimately, this approach will serve as a basis for developing more effective strategies aimed at mitigating the risk of contracting and transmitting AI among poultry farms in Nigeria, thus reducing the socioeconomic impact of the disease on the poultry value chain.

## 4. Conclusions

The findings of this study not only reveal that LPAI H3N8 and H5N2 viruses presently circulate in exotic and local turkey flocks in Oyo and Osun states, southwest Nigeria, but also emphasize the importance of routine surveillance for AIVs in different avian species as part of an early warning plan for the prevention of AI outbreaks in Nigeria. We advocate that farmers be educated on the dangers inherent in the practice of free-range rearing of birds as well as raising of different avian species together or in close proximity with other animals. This knowledge will help eliminate farm practices that could otherwise have enhanced the spread or interspecies transmission of AIVs in the country. Lastly, the use of social network and market chain analyses for future AI surveillance investigations is proposed as a basis for developing more effective AI control strategies.

## Figures and Tables

**Table 1:** Prevalence of avian influenza virus antibodies in sampled turkeys.

	Number sampled	Number positive (%)
State		
Oyo	315	19 (6.0)
Osun	95	4 (4.2)
Ondo	110	0 (0)
Management system		
Free-range	7	2^*∗*^ (28.6)
Intensive	513	21^*∗*^ (4.1)
Breed		
Exotic	189	10 (5.3)
Local	331	13 (3.9)
Age		
Grower	188	5 (2.7)
Adult	332	18 (5.4)
Sex		
Male	203	8 (3.9)
Female	317	15 (4.7)

^*∗*^Statistically significant.

*p* value = 0.034, OR = 9.4 (95% CI: 1.7–51.0).

**Table 2:** ELISA-positive sera and their HI antibody titres.

Sample number	State	Management system	Breed	Age	Sex	H3N8 HI titre	H5N2 HI titre
1	Oyo	Intensive	Exotic	Adult	Male	64	64
2	Oyo	Intensive	Local	Adult	Female	64	2048
3	Oyo	Free-range	Local	adult	Female	16	2048
4	Oyo	Free-range	Local	Adult	Female	8	0
5	Oyo	Intensive	Exotic	Adult	Male	16	2048
6	Oyo	Intensive	Exotic	Adult	Female	32	0
7	Oyo	Intensive	Local	Grower	Female	16	0
8	Oyo	Intensive	Exotic	Adult	Male	16	0
9	Oyo	Intensive	Exotic	Adult	Male	0	0
10	Oyo	Intensive	Exotic	Adult	Female	0	0
11	Oyo	Intensive	Exotic	Adult	Female	8	0
12	Osun	Intensive	Local	Grower	Female	32	0
13	Osun	Intensive	Local	Adult	Male	0	0
14	Osun	Intensive	Local	Grower	Female	0	0
15	Osun	Intensive	Local	Adult	Male	0	0
16	Oyo	Intensive	Local	Adult	Male	32	0
17	Oyo	Intensive	Local	Adult	Male	8	0
18	Oyo	Intensive	Exotic	Adult	Female	8	0
19	Oyo	Intensive	Exotic	Adult	Female	32	0
20	Oyo	Intensive	Local	Adult	Female	32	0
21	Oyo	Intensive	Local	Adult	Female	2048	0
22	Oyo	Intensive	Local	Grower	Female	2048	0
23	Oyo	Intensive	Local	Adult	Female	2048	0
